# Supplementary material for: Multi‐dataset identification of innovative feature genes and molecular mechanisms in keratoconus
Source: J Cell Mol Med. 2024 Sep 19;28(18):e70079. doi: 10.1111/jcmm.70079 (PMC11412914; doi:10.1111/jcmm.70079)
Supplement: Supplementary file 2 — Material S2. [file JCMM-28-e70079-s002.zip › 7.GSEA analysis/1.ARL11/KEGG_HYPERTROPHIC_CARDIOMYOPATHY_HCM.html]

Details for gene set KEGG\_HYPERTROPHIC\_CARDIOMYOPATHY\_HCM[GSEA]

|  || Dataset | ARL11.group.cls#h\_versus\_l |
| Phenotype | group.cls#h\_versus\_l |
| Upregulated in class | h |
| GeneSet | KEGG\_HYPERTROPHIC\_CARDIOMYOPATHY\_HCM |
| Enrichment Score (ES) | 0.23745464 |
| Normalized Enrichment Score (NES) | 0.8051024 |
| Nominal p-value | 0.7871901 |
| FDR q-value | 1.0 |
| FWER p-Value | 1.0 |
Table: GSEA Results Summary

  

Fig 1: Enrichment plot: KEGG\_HYPERTROPHIC\_CARDIOMYOPATHY\_HCM      
 Profile of the Running ES Score & Positions of GeneSet Members on the Rank Ordered List

  

| SYMBOL | TITLE | RANK IN GENE LIST | RANK METRIC SCORE | RUNNING ES | CORE ENRICHMENT || 1 | TNNI3 | na | 44 | 0.763 | 0.0723 | Yes |
| 2 | TNNT2 | na | 262 | 0.553 | 0.1195 | Yes |
| 3 | ITGA4 | na | 272 | 0.550 | 0.1723 | Yes |
| 4 | CACNB1 | na | 1069 | 0.381 | 0.1861 | Yes |
| 5 | CACNG8 | na | 1424 | 0.344 | 0.2091 | Yes |
| 6 | CACNA1F | na | 1554 | 0.333 | 0.2375 | Yes |
| 7 | TTN | na | 3142 | 0.234 | 0.2143 | No |
| 8 | ACTC1 | na | 5327 | 0.156 | 0.1663 | No |
| 9 | TPM2 | na | 5376 | 0.155 | 0.1799 | No |
| 10 | CACNA2D4 | na | 6234 | 0.131 | 0.1678 | No |
| 11 | IL6 | na | 6613 | 0.121 | 0.1686 | No |
| 12 | ITGB6 | na | 7342 | 0.105 | 0.1577 | No |
| 13 | TGFB2 | na | 7601 | 0.100 | 0.1599 | No |
| 14 | CACNG5 | na | 8337 | 0.086 | 0.1469 | No |
| 15 | MYH6 | na | 8587 | 0.081 | 0.1476 | No |
| 16 | CACNA2D3 | na | 8962 | 0.075 | 0.1441 | No |
| 17 | ITGA10 | na | 9425 | 0.068 | 0.1373 | No |
| 18 | ATP2A2 | na | 9649 | 0.065 | 0.1372 | No |
| 19 | ITGB7 | na | 10543 | 0.053 | 0.1165 | No |
| 20 | TPM1 | na | 10619 | 0.052 | 0.1194 | No |
| 21 | PRKAA2 | na | 11235 | 0.045 | 0.1060 | No |
| 22 | TPM4 | na | 11422 | 0.042 | 0.1047 | No |
| 23 | DAG1 | na | 11829 | 0.038 | 0.0967 | No |
| 24 | ITGB4 | na | 11987 | 0.036 | 0.0956 | No |
| 25 | ACTB | na | 12961 | 0.027 | 0.0702 | No |
| 26 | ACE | na | 14186 | 0.017 | 0.0365 | No |
| 27 | LMNA | na | 14922 | 0.012 | 0.0164 | No |
| 28 | PRKAG2 | na | 15005 | 0.011 | 0.0151 | No |
| 29 | MYBPC3 | na | 15364 | 0.008 | 0.0056 | No |
| 30 | EMD | na | 15650 | 0.006 | -0.0020 | No |
| 31 | PRKAG3 | na | 16733 | -0.002 | -0.0331 | No |
| 32 | MYH7 | na | 16998 | -0.003 | -0.0404 | No |
| 33 | PRKAA1 | na | 17397 | -0.006 | -0.0513 | No |
| 34 | PRKAB2 | na | 17459 | -0.006 | -0.0524 | No |
| 35 | DES | na | 17908 | -0.010 | -0.0644 | No |
| 36 | ITGA9 | na | 18231 | -0.012 | -0.0726 | No |
| 37 | IGF1 | na | 18329 | -0.013 | -0.0742 | No |
| 38 | TPM3 | na | 18375 | -0.013 | -0.0742 | No |
| 39 | CACNG3 | na | 18568 | -0.014 | -0.0784 | No |
| 40 | ITGA2 | na | 19176 | -0.018 | -0.0942 | No |
| 41 | ITGA3 | na | 19278 | -0.019 | -0.0953 | No |
| 42 | CACNB4 | na | 19492 | -0.020 | -0.0995 | No |
| 43 | CACNA1D | na | 19640 | -0.021 | -0.1016 | No |
| 44 | ITGA2B | na | 19828 | -0.023 | -0.1049 | No |
| 45 | ACTG1 | na | 20219 | -0.025 | -0.1137 | No |
| 46 | ITGA6 | na | 22097 | -0.041 | -0.1639 | No |
| 47 | CACNB2 | na | 22149 | -0.041 | -0.1614 | No |
| 48 | CACNG1 | na | 22207 | -0.042 | -0.1590 | No |
| 49 | PRKAG1 | na | 22360 | -0.044 | -0.1592 | No |
| 50 | DMD | na | 22362 | -0.044 | -0.1550 | No |
| 51 | TNF | na | 22702 | -0.047 | -0.1602 | No |
| 52 | ITGAV | na | 23305 | -0.053 | -0.1724 | No |
| 53 | CACNG2 | na | 23379 | -0.054 | -0.1693 | No |
| 54 | PRKAB1 | na | 23593 | -0.057 | -0.1699 | No |
| 55 | SGCD | na | 23777 | -0.060 | -0.1695 | No |
| 56 | TNNC1 | na | 24398 | -0.068 | -0.1808 | No |
| 57 | SGCB | na | 25142 | -0.078 | -0.1947 | No |
| 58 | CACNA1C | na | 25293 | -0.080 | -0.1914 | No |
| 59 | TGFB1 | na | 25459 | -0.082 | -0.1882 | No |
| 60 | ITGA7 | na | 26050 | -0.091 | -0.1964 | No |
| 61 | MYL3 | na | 26146 | -0.093 | -0.1902 | No |
| 62 | CACNA2D2 | na | 26358 | -0.096 | -0.1871 | No |
| 63 | ITGA11 | na | 26468 | -0.098 | -0.1808 | No |
| 64 | CACNB3 | na | 26714 | -0.103 | -0.1779 | No |
| 65 | ITGB5 | na | 27213 | -0.113 | -0.1814 | No |
| 66 | CACNG7 | na | 27851 | -0.127 | -0.1876 | No |
| 67 | ITGB1 | na | 28114 | -0.134 | -0.1822 | No |
| 68 | SLC8A1 | na | 29232 | -0.164 | -0.1986 | No |
| 69 | ITGB3 | na | 29449 | -0.171 | -0.1884 | No |
| 70 | ITGB8 | na | 29484 | -0.172 | -0.1728 | No |
| 71 | CACNG4 | na | 29566 | -0.174 | -0.1583 | No |
| 72 | CACNA2D1 | na | 29712 | -0.179 | -0.1453 | No |
| 73 | ITGA8 | na | 29843 | -0.183 | -0.1314 | No |
| 74 | ITGA1 | na | 29976 | -0.187 | -0.1172 | No |
| 75 | MYL2 | na | 30743 | -0.212 | -0.1188 | No |
| 76 | SGCG | na | 31057 | -0.222 | -0.1064 | No |
| 77 | LAMA2 | na | 31294 | -0.231 | -0.0908 | No |
| 78 | CACNG6 | na | 31816 | -0.253 | -0.0815 | No |
| 79 | SGCA | na | 32440 | -0.283 | -0.0722 | No |
| 80 | ITGA5 | na | 32559 | -0.291 | -0.0474 | No |
| 81 | TGFB3 | na | 33262 | -0.342 | -0.0347 | No |
| 82 | RYR2 | na | 33835 | -0.395 | -0.0131 | No |
| 83 | CACNA1S | na | 33887 | -0.403 | 0.0243 | No |
Table: GSEA details [plain text format]

  

Fig 2: KEGG\_HYPERTROPHIC\_CARDIOMYOPATHY\_HCM      
 Blue-Pink O' Gram in the Space of the Analyzed GeneSet

  

Fig 3: KEGG\_HYPERTROPHIC\_CARDIOMYOPATHY\_HCM: Random ES distribution      
 Gene set null distribution of ES for **KEGG\_HYPERTROPHIC\_CARDIOMYOPATHY\_HCM**
